# Yoga management of breast cancer-related lymphoedema: a randomised controlled pilot-trial

**DOI:** 10.1186/1472-6882-14-214

**Published:** 2014-07-01

**Authors:** Annette Loudon, Tony Barnett, Neil Piller, Maarten A Immink, Andrew D Williams

**Affiliations:** 1 Centre for Rural Health, University of Tasmania, Launceston, Australia; 2 School of Medicine, Flinders University, Adelaide, Australia; 3 School of Health Sciences, University of South Australia, Adelaide, Australia; 4 School of Health Sciences, University of Tasmania, Launceston, Australia

**Keywords:** Yoga, Breast cancer-related lymphoedema, Symptoms, Quality of life, Randomised controlled trial

## Abstract

**Background:**

Secondary arm lymphoedema continues to affect at least 20% of women after treatment for breast cancer requiring lifelong professional treatment and self-management. The holistic practice of yoga may offer benefits as an adjunct self-management option. The aim of this small pilot trial was to gain preliminary data to determine the effect of yoga on women with stage one breast cancer-related lymphoedema (BCRL). This paper reports the results for the primary and secondary outcomes.

**Methods:**

Participants were randomised, after baseline testing, to receive either an 8-week yoga intervention (n = 15), consisting of a weekly 90-minute teacher-led class and a 40-minute daily session delivered by DVD, or to a usual care wait-listed control group (n = 13). Primary outcome measures were: arm volume of lymphoedema measured by circumference and extra-cellular fluid measured by bioimpedance spectroscopy. Secondary outcome measures were: tissue induration measured by tonometry; levels of sensations, pain, fatigue, and their limiting effects all measured by a visual analogue scale (VAS) and quality of life based on the Lymphoedema Quality of Life Tool (LYMQOL). Measurements were conducted at baseline, week 8 (post-intervention) and week 12 (four weeks after cessation of the intervention).

**Results:**

At week 8, the intervention group had a greater decrease in tissue induration of the affected upper arm compared to the control group (p = 0.050), as well as a greater reduction in the symptom sub-scale for QOL (p = 0.038). There was no difference in arm volume of lymphoedema or extra-cellular fluid between groups at week 8; however, at week 12, arm volume increased more for the intervention group than the control group (p = 0.032).

**Conclusions:**

An 8-week yoga intervention reduced tissue induration of the affected upper arm and decreased the QOL sub-scale of symptoms. Arm volume of lymphoedema and extra-cellular fluid did not increase. These benefits did not last on cessation of the intervention when arm volume of lymphoedema increased. Further research trials with a longer duration, higher levels of lymphoedema and larger numbers are warranted before definitive conclusions can be made.

## Background

Breast cancer is the second most common form of cancer in women after skin cancer, and its incidence is expected to rise due to the ageing population [1]. In spite of improvements to surgical and radiotherapy treatment, at least 20% of treated women will continue to be diagnosed with breast cancer-related lymphoedema (BCRL) [2] that requires lifelong treatment and management [3].

As well as fibrosis of the tissue and increased risk of infection [4], women with BCRL can experience a range of debilitating sensations and symptoms [5,6] and in comparison to women who have had breast cancer treatment without lymphoedema, have impaired physical function [7] and lower quality of life (QOL) [8].

Due to the multi-dimensional consequences of BCRL, a holistic management approach, including exercise, is recommended [3]. Previously, exercise participation was discouraged due to concerns that exercise may exacerbate the condition; however, there is growing evidence to support the use of progressive and supervised exercise for women with BCRL with adequate warm-up, cool-down, appropriate rests [9] and suitable training of staff [10]. This evidence comes from studies covering a range of exercise modalities, which have reported no increase in severity [11,12] and fewer exacerbations of lymphoedema [13], increased strength [13], reductions in symptoms [13,14], and improvements in QOL [15,16]. In light of this, research into other holistic interventions, including yoga, for women with BCRL has been recommended [6,9,14]. Yoga is a holistic system of practices that aims to create balance in the physical, mental and emotional self [17]. It includes breathing (pranayama), postures (asana), meditation and relaxation. The physical movements and slow breathing, which can be adapted to principles of lymphatic clearing, have been used effectively as part of a holistic treatment to reduce lower limb lymphoedema from filariasis [18]. The practices of breathing, meditation and relaxation have been reported to improve the psychosocial functioning of women during and after breast cancer treatment [19]. These outcomes may be transferrable to women with BCRL. Women with BCRL are already attending yoga sessions [20], though the outcomes from this have not been systematically investigated.

The aim of this study was to obtain preliminary data to determine the effect of yoga on women with BCRL. This paper reports the results of the primary outcomes of lymphoedema status and the secondary outcomes of lymphoedema symptoms and QOL in women with stage one BCRL. We hypothesised that yoga would reduce swelling caused by lymphoedema relative to usual care and would lead to a reduction in tissue induration and severity of sensations, pain and fatigue associated with lymphoedema and their limiting effects, and improve QOL.

## Methods

### Study design

The trial was a multi-centred randomised controlled pilot trial using a parallel design with participants allocated to intervention or control on a 1:1 allocation ratio. The study was registered with the Australian New Zealand Clinical Trials Registry and ethics approval was granted by the University of Tasmania’s Social Sciences Human Research Ethics Committee. This study was part of a larger mixed methods study. The full protocol has been described previously [21]. This paper presents the results for the primary outcomes of arm volume of lymphoedema and extra-cellular fluid and the secondary outcomes of tissue induration, sensations and their limiting effects and QOL.

After meeting the selection criteria and giving informed consent, participants were randomised to a yoga intervention group or a wait-listed usual care control group. Outcome measurements were performed at baseline (week 0), week 8 (on completion of intervention) and at week 12 (one month after intervention). All intervention and data collection sessions were held in Community Health Centres at two locations, Hobart and Launceston, between February and May 2011. Both locations consisted of an intervention and control group.

Randomisation was conducted by a person not associated with the trial from a computer-generated random number system (http://www.randomization.com). No stratification occurred. Participants received notification of their group allocation in sealed envelopes after baseline testing.

### Participants

#### *Eligibility criteria*

Women were eligible for inclusion if they had stage one unilateral secondary lymphoedema of the arm, as defined by the International Society of Lymphology [3] confirmed by a professional lymphoedema therapist, and had completed treatment for breast cancer (surgery, radiotherapy and chemotherapy) at least six months previously, were over 18 and had sufficient English literacy to provide informed consent. Briefly, stage one lymphoedema is defined as early stage lymphoedema that will subside with elevation and may have signs of pitting and was chosen for this trial for two principal reasons. Firstly, as this was the first 8-week pilot trial to use a yoga intervention, women’s safety was paramount and it was thought that lower levels of lymphoedema may be more stable during the intervention period. Secondly, the standard offers a definition that could be confirmed or negated by a qualified lymphoedema therapist to account for different methods of clinical measurement. Measurement of women to confirm lymphoedema status was not possible prior to baseline testing because non-metric measurement equipment was not available to clinicians.

Women were excluded if they had recurrent cancer, an infection, were having Complex Lymphoedema Therapy, if they were pregnant, wore a pacemaker, which would affect bioimpedance spectroscopy (BIS) readings, or had severe psychological illness. All participants in the trial received a manual containing information on best current care for BCRL. Women were advised not to change current exercise nor commence any new activity during the study period and to seek immediate medical help if they experienced an exacerbation of lymphoedema during the trial.

#### *Control group*

Participants randomised to the control group maintained their usual self-care as advised by their lymphoedema therapist. Self-care included wearing of compression sleeves, self-massage, skin protection and continued usual lymphatic treatment. The control group were offered yoga classes at the completion of the final measurement.

#### *Yoga intervention group*

Participants randomised to the intervention group attended a weekly 90-minute yoga class taught by an experienced and accredited yoga teacher with qualifications in yoga therapy and Manual Lymphatic Drainage. Participants were also given a DVD with a 45-minute yoga session and instructed to perform it daily. The DVD followed the same sequence of practices as the class, with fewer postures and a shorter relaxation. Participants received a logbook in which they recorded their daily practice along with any relevant comments. Women were given the choice to wear a compression sleeve and, if removed, instructed to wear it again immediately after the yoga session [22].

The yoga session included documented breathing practices, physical postures, meditation and relaxation techniques according to the Satyananda Yoga® tradition [23] (Table 1). This style offers systematised practices and instruction thought to be suited to women with BCRL. Options for modifications were offered in the class and DVD. The practices were chosen to promote lymphatic drainage and to reduce stress and conformed with exercise guidelines and precautions for women with BCRL [3,9]. A full rationale for the session and chosen practices has been provided previously [24].

**Table 1:** Yoga session weeks 1–8 including on DVD

**Practice and time allocated**	**Sanskrit**
**Settling and Breathing 10 minutes (DVD 10 minutes)**	
Settling with awareness^*^	Kaya Sthairyam [1]
Mindfulness (inner silence)^*^	Antar Mouna level one [2]
Abdominal, thoracic, clavicular breath^*^	Pranayama [3]
Full yoga breath^*^	Pranayama [3]
**Postures 35 minutes (DVD 25 minutes)**[3]	
1a Neck turns^*^	Greeva Sanchalana
1b Add outward rotation of opposite arm^*^	Utthanpadasana-variation
3 Knee hugs-leg lock pose^*^	Supta Pawanmuktasana
4 Shoulder circles^*^	Shandha Chakra
5 Bent arm opening, chest towards knees^*^	Naukasana-variation combined with Namaskarasana-variation of arms
6 Lying Archer^*^	Akarna Dhanurasana-variation
7 Lying rotation^*^	Supta Udarakarshanasana-variation
8 Arm/leg stretch^*^	Supta Pawanmuktasana
9 Sitting rowing^*^	Nauka Sanchalanasana
10 Standing archer^*^	Akarna Dhanurasana
11 Modified rope climbing^*^	Rajju Karshanasana-variation
12 Modified arm raise, knee bend^*^	Tadasana-variation
13 Modified side bend^*^	Trikonasana-variation
14 Standing rotation^*^	Kati Chakrasana
15 Standing Cat^*^	Marjari-asana_variation
16 Modified one legged prayer balance^*^	Eka Pada Pranamasana
17 Sitting neck turns^*^	Greeva Sanchalana
**Mindfulness, Pranayama, Meditation 10 minutes**	
Settling with awareness and stillness	Kaya Sthairyam
^*^Mindfulness practice (inner silence)	Antar Mouna level one Weeks 1-4
	Antar Mouna level one Weeks 5-8
Alternate nostril breathing	Nadi Shodan [3]
^*^Visualisation One-pointed focus-lymph system	Dharana [2]
Meditation One-pointed focus candle	Tratak [3] Weeks 7 and 8
**Relaxation-meditation 20 minutes (DVD 10 minutes)**	
^*^Deep relaxation	Yoga Nidra [4]
**Discussion yoga themes 10 minutes**	

^*^Practice in DVD.

Full description of each practice:

1. Saraswati, N., Dharana Darshan-Yogic, Tantric and Upanishadic Practices of Concentratrionand Visualization. 2003. Yoga Publications Trust, Munger, Bihar, India. 439.

2. Saraswati, S. Meditations from the Tantras. 2001. Yoga Publications Trust, Munger, Bihar, India. 367.

3. Saraswati, S. Asana, Pranayama, Mudra, Bandha. 1996, Munger, India: Bihar School of Yoga. 543.

4. Saraswati, S. Yoga Nidra. 6th ed. 2006, Munger, India: Bihar School of Yoga. 261.

### Outcome measures

Measurements, based on validated instruments and protocols, were taken by trained researchers blinded to the group allocation and previous results. Severity of lymphoedema for arm volume and extra-cellular fluid and tissue induration were measured by an experienced and registered lymphoedema physiotherapist at each location. Inter-rater reliability between lymphoedema therapists was assessed pre-trial and rated as acceptable for variability’s between 2-3%. Other trained assessors administered anthropometric measurements and questionnaires at both places on different days. To ensure consistency at each time-point, participants attended at the same time throughout the trial and the same assessor was responsible for each measure. Participants were requested to abstain from alcohol for 12 hours and caffeine and exercise for two hours before testing to increase the validity of the BIS readings.

On arrival at the testing facility, participants underwent anthropometric measurements wearing light clothes and no footwear. Participants then underwent measures of lymphoedema and tissue induration and completed VAS and QOL questionnaires in the same order at each measurement session.

#### *Lymphoedema*

Lymphoedema was measured with the participant supine and arm dominance noted. Women removed their sleeve on entering the measurement venue. Two measures were chosen: arm volume from circumference and extra-cellular fluid from BIS. These measures can give different outcomes [25,26]; for example, BIS results include the fluid in the upper part of the arm where it is difficult to get circumferential readings.

Circumferential readings were taken by a Jobst non-stretch tape according to an established protocol [27] at the metacarpophalangeal joint and at 10 cm intervals from the styloid process. Volume of arm lymphoedema was calculated using the truncated cone formula [28] from the addition of circumferential readings using an Excel spreadsheet that compared the affected to the non-affected arm, resulting in a measure of Absolute Arm Volume between the affected and non-affected arm. Measurements were recorded in millilitres (ml).

Extra-cellular fluid was measured by BIS L-dex XCA™ (Bio-Impedimed, Queensland) [29]. Electrodes were placed at anatomical landmarks at the wrist of each arm and right ankle to provide a low-frequency electrical current. An increase in extra-cellular fluid is paralleled by a decrease in impedance and the result recorded as a ratio to the non-affected arm, taking into consideration arm dominance [26]. The result was an L-dex reading, calculated from software provided by the manufacturer.

#### *Tissue induration*

Induration of fibrotic tissue was measured using a digital tonometer, model 1383 (Biomedical Engineering, Flinders Medical Centre, South Australia) [30]. The digital tonometer measures the resistance to compression in the superficial tissues at a given point on the areas of lymph drainage (lymphatic territory) [25]. Measurements were taken 10 cm from the cubital fossa on the forearm and 10 cm from the cubital fossa on the upper arm, in the middle of the areas of lymphatic territory. Anterior trunk measurements were taken at the mid-clavicular line between the second and third ribs and at the posterior trunk between the acromion and the first thoracic rib in the subscapular fossa. Measurements were taken three times at each position, separated by a three-second pause, for the affected and non-affected arm and trunk. A higher score denoted a higher level of induration of fibrotic tissue. The average of three measurements was recorded in millimetres (mm).

#### *Sensations, pain and fatigue and their limiting effects*

Participants recorded the severity of sensations, pain and fatigue, and the degree to which sensations, pain and fatigue limited activity on the day of measurement on a 10 cm Visual Analogue Scale (VAS) [31]. A score of 0 cm indicated “no discomfort” and a score of 10 cm indicated “the worst imaginable”.

#### *Quality of life*

A validated questionnaire, developed specifically to measure QOL for people with arm lymphoedema, LYMQOL [32], was used. Total QOL was self-recorded with scores from 0–10, ten being the best and zero the worst rating on the day of testing. Sub-scales, each consisting of several questions, for function, symptoms, appearance and emotions, were also self-recorded. Each question was scaled from 1 to 4, four being the worst. The score for each sub-scale was based on the mean of the ratings for sub-scale related questions. A higher score indicated a lower QOL rating for that sub-scale.

#### *Data analysis*

An a priori sample size calculation was performed and indicated that 19 participants per group would be required to detect statistically significant changes in the primary outcome variables. As the number of participants was limited by time and other practical constraints, a pilot study was conducted.

Baseline information between treatment groups for demographic and medical characteristics were compared by independent two-tailed t-tests for continuous variables and by Yates corrected chi-square tests for categorical variables (SPSS version 19; IBM, Armonk, New York, USA). Statistical analyses of outcome measures at baseline and changes between groups at weeks 8 and 12 were performed using STATA statistical software (version 12; STATA Corporation, College Station, Texas, USA). Parametric longitudinal data were analysed via mixed methods linear regression (ANOVA). Where assumptions of linear regression were violated, data were analysed using non-parametric analysis via ordinal logistic regression. In both methods of analysis, the independent variables were time and group while the dependent variables included lymphoedema (L-Dex, arm volume), tissue induration, sensations and quality of life. Post-hoc testing was performed on all data using the Holms test to locate the means that were significantly different. Statistical significance was set at p < 0.05. Due to the low sample size neither multivariate nor covariate analyses were performed. Data is presented as Mean and Standard Deviation unless otherwise indicated.

## Results

### Participant flow and compliance

**Figure 1:**
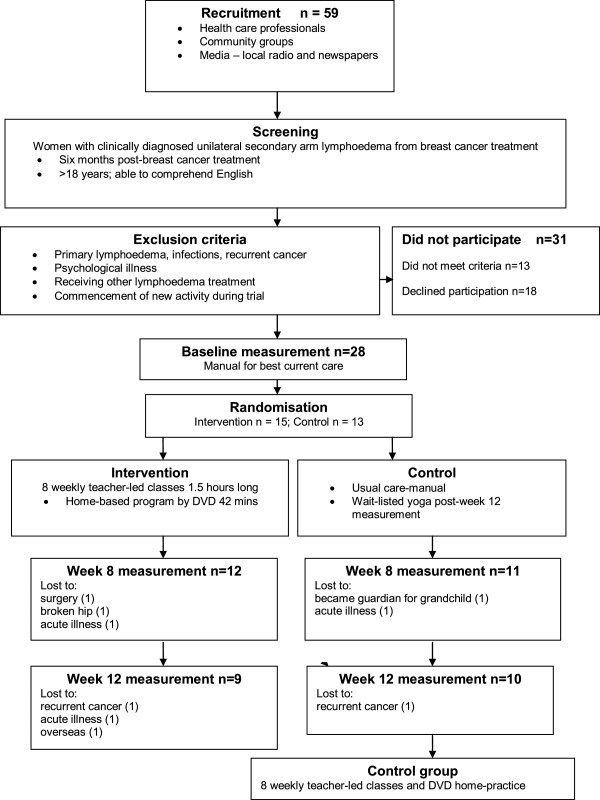
Trial flow.

The flow of participants through the trial is outlined in Figure 1. Participants in the study were asked at each assessment point whether they had experienced any adverse events (pain or abnormal sensation) that might be associated with the intervention. There were no adverse events attributable to either the yoga or the control intervention. Two participants withdrew after being diagnosed with recurrent cancer during the trial while five others experienced adverse events requiring their withdrawal from the study that were unrelated to either their condition or the treatment. Details of these adverse events are included in Figure 1.

Due to attrition, fewer participants returned for the week-12 follow-up than completed the week 8 measurements. Consequently, the results from baseline to end of the 8 week intervention (b-8) and end of the week 8 intervention to week 12 follow up (8b-12) were analysed separately. Attendance at the group yoga sessions was high (97%), as was self-reported compliance with the home-practice DVD (86%).

### Baseline demographics and medical characteristics

Twenty-three participants, with a mean age of 57.6 ± 10.5 years (range 34–80) and a mean BMI of 27.2 ± 4.9 kg/m^2^ (range 20.4-37.3), completed the eight week intervention and 19 women returned for the week 12 follow up measurement. No women in either group were doing yoga, apart from that prescribed for the intervention group, during the trial, nor had they done it since developing BCRL.

At baseline, the intervention group had a significantly higher BMI (29.1 kg/m^2^ ± 4.6; p = 0.023) than the control group (25.1 kg/m^2^ ± 4.5). There were no other significant differences between the intervention and control groups in demographics or medical characteristics at baseline (Table 2).

**Table 2:** Baseline demographics and medical characteristics of the groups

**Characteristics**	**Intervention (n = 12)**	**Cotnrol (n = 11)**	**P value**^**a**^
	**Mean ± SD**	**Mean ± SD**	
Age (years)	55.1 ± 2.5	60.5 ± 3.6	0.230
BMI (kg/m^2^)	29.1 ± 4.6	25.1 ± 4.5	0.023
Range	36-65	34-80	
Number nodes removed	14.3 ± 2.3	11.2 ± 2.7	0.429
Number of positive nodes	1.5 ± 0.5	3.7 ± 2.3	0.321
How long lymphoedema (years)	4.9 ± 1.6	5.1 ± 1.9	0.900
How long post-surgery	1.2 ± 0.4	1.9 ± 0.7	0.822
	**n(%)**	**n(%)**	**P value**^**b**^
**Living arrangements**			
Live alone	2(14)	3(27)	0.912
Live with others	10(83)	8(73)	
**Employment**			
Home, retired	5(42)	8(73)	0.280
Employed	7(58)	3(27)	
Fitness (self-scored)			
Low	2(17)	1(9)	
Medium	8(67)	8(73)	0.913
High	2(17)	2(18)	
**Breast cancer surgery**			
Lumpectomy	5(42)	3(27)	0.882
Mastectomy	7(58)	8(73)	
**Type of lymph dissection**			
Sentinel node	0	1(9)	0.980
Axillary clearance	12(100)	10(91)	
**Stage of breast cancer**			
DCIS	0	1(9)	0.976
1	3(25)	4(36)	
2	6(50)	5(45)	
3	3(25)	1(9)	
**Treatment and effects**			
Chemotherapy	8(66)	6(54)	0.867
Effects chemotherapy	7(58)	4(36)	0.525
Radiotherapy	9(75)	7(64)	0.890
Effects radiotherapy	8(67)	3(27)	0.198
**Most common area of radiotherapy**			
Chest	7(58)	7(64)	0.909
Axilla	2(17)	2(18)	
Axilla and chest	3(25)	2(18)	
**Other post-surgery effetcs**			
Post-surgery infection	3(25)	3(27)	0.725
Post-surgery cording	3(25)	3(27)	0.725
Post-surgery fluid removal	9(75)	9(82)	0.913

### Outcomes

#### *Lymphoedema status*

All participants had been clinically diagnosed with lymphoedema by professional and experienced lymphoedema therapists. We used two methods of measure to determine changes in lymphoedema in the trial due to the variance that can occur in the definition of lymphoedema and to quantify changes that could occur from the intervention. At some measurements some women in both groups may have had variation in lymphoedema status. As this was a controlled trial we did not account for fluctuation.

#### *Volume of arm lymphoedema*

There was no between group difference in volume of arm lymphoedema measured by circumference at baseline. From b-8, there was no significant change between groups (Table 3).

**Table 3:** Results lymphoedema and tissue induration

	**Group mean ± SD b-8**		**Between group changes 0-8**			**Group mean ± SD 8b-12**		**Between group changes 8b-12**	
**Variable Gp(n)**	**Week 0 M ± SD**	**Week 8 M ± SD**	**∆int-∆con 0–8 MD; (95% Cl)**	**P(0–8)**	**Variable Gp(n)**	**Week 8b M ± SD**	**Week 12 m ± SD**	**∆int-∆con 8b-12 MD; (95% CI)**	**P(8b-12)**
**Lymphoedema** L-dex (BIS)									
Con (11)	6.49 ± 14.14	7.66 ± 12.64	-1.03; (-4.17 to 2.10)	0.519	Con(10)	8.68 ± 12.83	7.83 ± 13.59	2.57; (-1.04 to 6.18)	0.163
Int(12)	5.89 ± 9.07	6.03 ± 8.24			Int(9)	4.96 ± 6.40	6.67 ± 7.08		
Arm volume									
Con(11)	59.89 ± 78.53	60.75 ± 80.69	-30.28; (-69.33 to 8.78)	0.129	Con(10)	67.65 ± 82.39	58.17 ± 100.42	35.20; (3.09 to 67.32)	0.032
Int(12)	101.45 ± 75.08	72.03 ± 80.77			Int(9)	60.82 ± 82.84	86.53 ± 78.29		
**Tissue induration (Tonometry)** Forearm affected									
Con(11)	13.96 ± 3.82	14.06 ± 4.80	0-1.89; (-4.88 to 1.16)	0.227	Con(10)	13.65 ± 4.85	12.45 ± 4.36	-0.43; (-2.77 to 1.92)	0.42
Int(12)	15.78 ± 4.79	14.02 ± 3.59			Int(9)	13.87 ± 3.68	12.26 ± 2.41		
Forearm non-affected									
Con(11)	15.61 ± 4.17	15.81 ± 5.20	-140; (-4.59 to 1.78)	0.387	Con(10)	15.37 ± 5.27	12.66 ± 4.00	0.10; (-3.09 to 3.30)	0.95
Int(12)	15.92 ± 5.51	14.72 ± 5.42			Int(9)	14.68 ± 6.23	12.07 ± 4028		
Upper arm affected									
Con(11)	10.66 ± 5.57	12.54 ± 5.91	-3.20; (-641 to 0)	0.050	Con(9)	12.08 ± 6.02	8.92 ± 5.19	0.95; (-1.40 to 3.30)	0.43
Int(12)	11.10 ± 6.09	9.77 ± 3.33			Int(9)	9.63 ± 3.53	7.42 ± 4.39		
Upper arm non-affected									
Con(10)	10.14 ± 4.42	12.05 ± 6026	-2.88; (-5.82 to 0.06)	0.055	Con(10)	11.46 ± 6.34	7.97 ± 5.18	0.53; (-1.98 to 3.03)	0.68
Int(12)	9.88 ± 4.09	8.91 ± 3.621			Int(9)	8.17 ± 3.54	5.21 ± 3.10		
Chest affected									
Con(11)	6.78 ± 2.39	5.53 ± 3.41	-0.36; (-2.63 to 1.91)	0.758	Cont(10Int(9)	5.22 ± 3.43	5.76 ± 1.50	-0.60; (-2.94 to 1.73)	0.61
Int(12)	6.34 ± 2.01	4.73 ± 1.75				4.23 ± 1.14	4.17 ± 1.09		
Chest non-affected									
Con(11)	5.59 ± 1.84	4.76 ± 2.65	-0.34; (-2.85 to 2.17)	0.878^†^	Con(10)	4.70 ± 2.79	6.18 ± 5.00	-1.22; (-5.33 to 2.90)	0.56
Int(12)	6.06 ± 1.87	4.90 ± 1.97			Int(9)	4.98 ± 2.23	5.25 ± 1.60		
Upper back affected									
Con(11)	16.17 ± 2.39	16.76 ± 4.32	0.19; (-3.77 to 4.16)	0.924	Con(10)	16.48 ± 4.45	13.21 ± 5.06	1.10; (-1.51 to 3.70)	0.41
Int(12)	16.06 ± 4.18	16.84 ± 4.82			Int(9)	15.81 ± 4.28	13.63 ± 2.930		
Upper back non-affected									
Con(11)	14.32 ± 3.87	15.13 ± 5.54	-1.42; (-4.85 to 2.02)	0.419	Con(10)	15.04 ± 5.79	14.43 ± 4.32	-0.48; (-3.90 to 2.95)	0.78
Int(12)	15.55 ± 4.21	15.05 ± 4.86			Int(9)	14.66 ± 5.33	13.58 ± 3.83		

∆ = change; Gp = Group; M ± SD = Mean ± Standard Deviation; MD = Mean Difference; CI = Confidence Interval.

n = number.

Con = control group; Int = intervention group.

^†^ = non-parametic analysis; b = week 0.

From 8b-12 (Table 3), there was a significant change between groups in volume of arm lymphoedema (p = 0.032) due to the significant increase in the intervention group (25.72 ml; 95% CI: 3.01 to 48.42; p = 0.026).

#### *Extra-cellular fluid*

There was no between group difference in extra-cellular fluid measured by BIS at baseline. From b-8 and 8b-12 there were no significant changes between groups (Table 3). Therefore, this pilot trial was negative with respect to the pre-specified primary outcomes.

#### *Tissue induration*

There were no between group differences for any measure of tissue induration of the affected or non-affected area measured by tonometry at baseline. From b-8, there was a significant decrease in tissue induration of the affected upper arm in the intervention compared to the control group (p = 0.050) (Table 3). From 8b-12, there were no significant changes in tissue induration between groups.

#### *Degree of sensations, pain, fatigue and their limiting effects*

There were no between group differences for degree of sensations, pain, fatigue and their limiting effects measured by the VAS scale at baseline nor from b-8 or 8b-12 (Table 4).

**Table 4:** Results sensations, pain, fatigue, and limiting effect of sensations, pain, fatigue

**VAS**	**Group mean ± SD b-8**		**Between group changes 0-8**			**Group mean ± SD 8b-12**		**Between group changes 8b-12**	
**Variable Gp(n)**	**Week 0 M ± SD**	**Week 8 M ± SD**	**∆int-∆con 0–8 MD; (95% CI)**	**P(0–8)**	**Variable Gp(n)**	**Week 8b M ± SD**	**Week 12 M ± SD**	**∆int-∆con 8b-12 MD; (95% CI)**	**P(8b-12)**
**Sensations**									
Con(11)	1.97 ± 1.89	2.01 ± 2.15	-0.55; (02.33 to 1.23)	0.345^†^	Con(10)	2.20 ± 2.17	2.20 ± 2.12	0.30; (-1.21 to 1.81)	0.698
Int(12)	2.39 ± 2.12	1.88 ± 1.83			Int(9)	1.96 ± 1.59	2.26 ± 2.29		
**Pain**									
Con(11)	1.69 ± 2.31	1.44 ± 2.24	0.06; (-0.74 to 0.87)	0.878	Con(10)	1.57 ± 2.31	1.16 ± 1.48	0.81; (-0.33 to 1.95)	0.165
Int(12)	0.99 ± 1.53	0.80 ± 1.48			Int(9)	1.00 ± 1.67	1.40 ± 1.84		
**Fatigue**									
Con(11)	1.71 ± 2.21	2.06 ± 2.52	-1.05; (-2.50 to 0.41)	0.117^†^	Con(10)	2.26 ± 2.56	1.57 ± 1.54	0.42; (-1.45 to 2.30)	0.551^†^
Int(12)	2.58 ± 2.60	1.88 ± 2.23			Int(9)	2.37 ± 2.50	2.10 ± 1.77		
**Sensations limit activity**									
Con(11)	1.35 ± 2.81	0.93 ± 1.90	-0.18; (-1.66 to 1.30)	0.793^†^	Con(10)	1.01 ± 1.98	0.8 ± 1.70	0.37; (-0.49 to 1.22)	0.399
Int(12)	1.43 ± 1.76	0.83 ± 0.74			Int(9)	1.20 ± 1.54	1.10 ± 1.54		
**Pain limit activity**									
Con(11)	0.57 ± 1.10	1.31 ± 2.39	-0.99; (-2.06 to 0.09)	0.362^†^	Con(10)	1.42 ± 2.49	0.89 ± 1.59	0.72; (-0.80 to 2.24)	0.353
Int(12)	0.81 ± 1.44	0.56 ± 0.58			Int(9)	0.61 ± 0.58	0.80 ± 0.93		
**Fatigue limit activity**									
Con(11)	0.69 ± 1.51	1.34 ± 2.57	-1.0-; (-2.44 to 0.27)	0.315^†^	Con(10)	1.46 ± 2.67	1.07 ± 1.74	0.61; (-0.78 to 2.00)	0.389
Int(12)	1.38 ± 1.85	0.93 ± 0.95			Int(9)	1.03 ± 0.95	1.26 ± 1.24		

∆ = change; Gp = Group; M ± SD = Mean ± Standard Deviation; MD = Mean Difference; CI = Confidence Interval; n = number.

Con = control group; Int = intervention group.

^†^ = non-parametric analysis.

B = week 0.

#### *Quality of life*

There were no between group differences for the sub-scales and total QOL score measured by LYMQOL at baseline. From b-8, there was a significant decrease (improvement) in the intervention compared to the control group in the QOL sub-scale of symptoms p = 0.038) (Table 5). From 8b-12, there were no significant changes between groups (Table 5).

**Table 5:** Results total Quality of Life (QOL) and QOL sub-scales

**QOL**	**Group mean ± SD b-8**		**Between group changes 0-8**			**Group mean ± SD 8b-12**		**Between group changes 8b-12**	
**Variable Gp(n)**	**Week 0 M ± SD**	**Week M ± SD**	**∆int-∆con 0–8 MD; (95% CI)**	**P(0–8)**	**Variable Gp(n)**	**Week 8b M ± SD**	**Week 12 M ± SD**	**∆int-∆con 8b-12 MD; (95% CI)**	**p(8b-12)**
**Total QOL**									
Con(11)	7.91 ± 1.22	7.45 ± 1.44	1.04; (-0.19 to 2.26)	0.437^†^	Con(10)	7.40 ± 1.51	7.40 ± 1.51	0.44; (-0.38 to 1.27)	0.290
Int(12)	6.83 ± 2.55	7.42 ± 1.24			Int(9)	7.33 ± 0.87	7.78 ± 1.09		
**Function**									
Con(11)	1.36 ± 0.40	1.30 ± 0.36	-0.13; (-0.34 to 0.09)	0.364^†^	Cont(10)	1.31 ± 0.38	1.35 ± 0.33	0.13; (-0.07 to 0.33)	0.210
Int(12)	1.48 ± 0.48	1.30 ± 0.31			Int(9)	1.34 ± 0.33	1.51 ± 0.14		
**Appearance**									
Con(11)	1.56 ± 0.81	1.56 ± 0.86	-0.07; (-0.34 to 0.20)	0.627	Con(10)	1.60 ± 0.69	1.60 ± 0.69	0.10; (-.25 to 0.45)	0.578
Int(11)	1.50 ± 0.34	1.43 ± 0.33			Int(9)	1.42 ± 0.37	1.52 ± 0.37		
**Symptoms**									
Con(11)	1.69 ± 0.37	1.82 ± 0.54	-0.44; (-0.74 tro -0.13)	0.038^†^	Con(10)	1.90 ± 0.49	1.73 ± 0.47	0.17; (-0.05 to 0.39)	0.124
Int(12)	2.11 ± 0.61	1.81 ± 0.40			Int(9)	1.91 ± 0.38	1.91 ± 0.40		
**Emotions**									
Con(11)	1.71 ± 0.56	1.61 ± 0.49	-0.18; (-0.62 to 0.26)	0.430	Con(10)	1.62 ± 0.52	1.60 ± 0.56	0.04; (-0.24 to 0.31)	0.801
Int(12)	1.86 ± 0.74	1.58 ± 0.7			Int(9)	1.44 ± 0.45	1.46 ± 0.43		

∆ = change; Gp = Group; M ± SD = Mean ± Standard Deviation; MD = Mean Difference; CI = Confidence Interval.

N = number.

Con = control group; Int = intervention group.

^†^ = non-parametric analysis.

0 = baseline.

## Discussion

The aim of this small pilot trial was to gain preliminary evidence on the effects of an 8-week yoga intervention on women with stage one BCRL. This paper reports the results of the primary and secondary outcomes. The swelling caused by lymphoedema did not decrease. Eight weeks of yoga resulted in reductions in tissue induration of the affected upper arm and in the QOL sub-scale of symptoms specific to lymphoedema. However, these improvements were not sustained at one month post-intervention when arm volume of lymphoedema increased.

Severity of lymphoedema did not decrease and is comparable to the response from exercise interventions of varying durations and modalities for women with BCRL [12,13,15]. In the current study, L-dex readings from BIS were virtually unchanged and consistent with those of a 12-week combined aerobic and resistance intervention [11], many of whose participants had low levels of lymphoedema, as in the current trial. In comparison to the BIS results, a significant decrease in volume of arm lymphoedema was recorded at week 8 in the intervention group. While it is possible this result may have been affected by a non-significant higher mean arm volume in the yoga group at baseline (Table 3), the result is consistent with the volume reduction (p = 0.07) found after a four-week daily tai-chi and breathing intervention [14]. In addition, in our study this result was reversed at the week 12 follow-up. These results suggest that yoga may be beneficial in reducing or at least *not increasing* volume of arm lymphoedema in women with early-stage BCRL but needs to be ongoing as the benefits may disappear when yoga ceases.

The reduction in tissue induration of the affected upper arm in the intervention group compared to the control was a significant beneficial outcome of this trial. The yoga intervention focussed on the repetition and coordination of physical movements based on range of motion of the shoulders, spine and whole body, leading to a gentle, rhythmic stretching and compression of the skin and underlying tissue, particularly in the arms, chest and upper back. Researchers in a tai-chi trial that used a gentle arm opening and closing exercise for women with BCRL and reported a significant reduction in the tissue induration of the chest (p = 0.005) [14] suggested that those actions may have reduced adhesions caused by fibrosis and improved the quality of the underlying connective tissue. Both the current and the tai-chi trial combined slow physical movement with slow and controlled breathing, which also may have created a gentle stretching of the connective tissue of the secondary muscles of breathing, such as the pectoral and serratus anterior muscles, perhaps softening the tissue and enabling less restriction of shoulder movement. As stage one lymphoedema may not be accompanied by actual fibrotic tissue [3] we are unable to confirm its use in reducing fibrotic tissue. However, as fibrosis of tissue is a debilitating effect of lymphoedema and can increase the possibility of infection, the outcomes from the tai-chi and the current trial offer preliminary evidence of the beneficial effects of tai-chi and yoga in softening tissue that warrant further research.

The yoga session offered in this trial consisted not only of physical practices with focussed awareness on the breath and body, but also specific practices of breathing, relaxation and meditation, which are considered effective in improving biopsychosocial functioning [33]. The reduction in the LYMQOL sub-scale of symptoms at the completion of the yoga intervention is perhaps indicative of the holistic beneficial effects of yoga. Symptoms of lymphoedema adversely affect physical function and QOL in women with BCRL [8,34]. A qualitative study into the effect of symptoms on the lives of women with BCRL [5] reported that ongoing symptoms continued to cause physical discomfort leading to emotional frustration and distress in spite of treatment to lessen the arm swelling. Moreover, persistent symptoms created continual challenges in the women’s daily life, led to an altered self-image and constantly reminded them of the breast cancer experience. In the current trial, the degree of sensations, pain and fatigue measured separately on the VAS scale did not decrease, and yet the QOL sub-scale of symptoms, consisting of a combination of six different symptoms, improved significantly, indicating a reduction in the adverse effect of symptoms on women’s QOL. A possible explanation for this combination of results could be that the actual symptoms did not reduce but the women’s reaction to them did. It is possible that such a finding may be attributable to the mindful awareness component of yoga, whereby practitioners learn to witness physical and mental discomfort without engaging with that discomfort. A study that used yoga as an intervention for women undergoing radiotherapy for breast cancer reported a positive correlation between a higher number of intrusive thoughts and benefit finding from the cancer experience [35]. The researchers suggested this may be a result of mindful awareness, in that the intrusive thoughts actually increased, but a negative reaction to the thoughts did not occur. Symptom management is integral to lymphoedema treatment [5,36]. Ridner and colleagues [6] postulated that current lymphoedema treatment may not deal adequately with the symptom cluster experienced by women with BCRL and so complementary therapies may provide other management options to reduce the adverse effects commonly reported. The results of the current trial support this postulation.

As other QOL sub-scales did not improve in the current trial, it may be that the reduction in symptoms precedes the improvement in other QOL domains. Comparison with other trials for women with BCRL is difficult, as different QOL tools were used that did not include a sub-scale of lymphoedema symptoms. Only one yoga trial could be found that reported on symptoms and QOL ratings. A 12-week yoga intervention for women during and after breast cancer treatment, using the Functional Assessment of Cancer Therapy QOL tool, found improvements in total QOL and in the social and emotional QOL sub-scales only in the women not experiencing symptoms from chemotherapy [37]. Although the symptoms experienced by women from chemotherapy are different to those from BCRL, it may indicate that symptom amelioration precedes improvement in other QOL domains. It would appear that a holistic yoga intervention may be effective in improving symptoms; however, a longer intervention with larger numbers is needed to further test the strength of this hypothesis or whether yoga leads to a decrease in the other QOL domains.

Physical therapy, as an early intervention, has been recommended for the prevention or reduction of the morbidity associated with the effects of breast cancer treatment and lymphoedema [9]. The yoga intervention was based on gentle and modified physical movements. The benefits gained may indicate that yoga can offer another option as an early physical therapy. It may also provide an intermediary to more strenuous forms of exercise and counteract the fear that some women have in resuming exercise [11,38]. Current best-practice recommends early intervention to prevent lymphoedema becoming worse [39]. That the generally lower levels of lymphoedema did not increase in this trial along with the reduction in tissue induration of the affected upper arm, and improvement in the symptoms sub-scale of QOL may offer preliminary support that yoga may be a helpful intervention post-surgery.

This trial had several limitations. Women in the current trial had been clinically diagnosed with stage one lymphoedema according to the definition from the International Society of Lymphology [3], using standardised testing and equipment and based on guidelines of the Australasian Lymphology Association [27]. However, many of the baseline scores for BIS (L-dex > 10) and volume of arm lymphoedema (>200 ml) were low and women with higher levels of BCRL may not have experienced similar results. Hence a recommendation for future studies would be to make an L-dex of >10 or volume of arm lymphoedema (>200 ml) an additional inclusion criteria and that this measure be confirmed by the research team. Furthermore, fluctuation of levels [7,40] may have occurred in the trial. However, it was considered the control group would account for any factors other than the intervention which might be responsible for changes in the outcome variables. As there were no significant within group differences in the control group for any measure of lymphoedema, fluctuation of levels seems an unlikely explanation for our results. There were large, albeit non-significant, mean differences between treatment groups in several variables at baseline. When baseline results were included as a covariate for comparison of the two groups for the primary outcome variables and those for which a statistically significant result was observed, this did not affect the significance of the result for the tested variable. As a result, it is unlikely that these imbalances affected changes occurring in the variables during the intervention period. In addition, sample size was small, which was partially due to all participants starting at the same time and yoga classes running as a group meaning that additional participants were unable to be recruited to make up for withdrawals and this may have limited some findings. A post-hoc power calculation has revealed that using this study as a pilot for a future study, 179 participants would be required per group aiming to detect a 12% difference in change in lymphoedema between the control and the intervention groups based on the observed standard deviation of the between group change as the standard deviation of the changes was greater than expected. A longer intervention may have increased the degree of change or the duration of the beneficial change. Further, the higher BMI of the yoga compared to the control group at baseline may have confounded the results, but due to the small sample size it was thought that covariate analysis using BMI would not produce substantial results. As BMI did not change over the course of the intervention, it was unlikely that it affected results. The yoga intervention was based on Satyananda Yoga® using specific practices thought to reduce the effects of lymphoedema and thus the findings may not be generalised to other styles of yoga.

## Conclusion

The outcomes of this small pilot trial provide preliminary evidence that an 8-week Satyananda Yoga® intervention, based on guidelines for exercise and lymphatic drainage did not exacerbate lymphoedema measured by arm volume and extra-cellular fluid and improved tissue induration of the affected upper arm and the QOL sub-scale of symptoms. However, the fact that these improvements were not maintained at the 1-month follow-up when arm volume of lymphoedema also increased suggests that the yoga needs to be ongoing. Future yoga trials of longer duration, higher levels of lymphoedema and higher participant numbers are warranted to better elucidate these potential benefits with the recommendation that future trials make an L-dex of >10 or volume of arm lymphoedema (>200 ml) an additional inclusion criteria confirmed by the research team.

## Abbreviations

BCRL: Breast cancer-related lymphoedema; QOL: Quality of life.

## Competing interests

The authors declare that they have no competing interests.

## Authors’ contributions

AL conceived the trial and with TB and ADW were responsible for the design of this trial and the construction of the measurement protocol. AL conducted the yoga intervention. NP was responsible for the design of the lymphoedema methodology. MAI assisted in design of the trial and advice for the yoga intervention. AL and ADW conducted the statistical analysis. AL and ADW drafted and TB, NP, MAI helped draft the manuscript. All authors read and approved the final manuscript.

## Authors’ information

AL is a yoga researcher and trains yoga teachers in yoga therapy. TB is Director of Rural Health, University of Tasmania. ADW is a senior lecturer in Clinical Exercise Science, University of Tasmania. NP is Director of the lymphoedema clinic at Flinders University. MAI is Director of physical movement and yoga researcher at University of South Australia.

## Pre-publication history

The pre-publication history for this paper can be accessed here:

http://www.biomedcentral.com/1472-6882/14/214/prepub
